# Sputum sample positivity for *Haemophilus influenzae* or *Moraxella catarrhalis* in acute exacerbations of chronic obstructive pulmonary disease: evaluation of association with positivity at earlier stable disease timepoints

**DOI:** 10.1186/s12931-021-01653-8

**Published:** 2021-02-24

**Authors:** Lucio Malvisi, Laura Taddei, Aparna Yarraguntla, Tom M. A. Wilkinson, Ashwani Kumar Arora, the AERIS Study Group

**Affiliations:** 1 GSK, Siena, Italy; 2 ICON, Chennai, India c/o GSK, Wavre, Belgium; 3 Faculty of Medicine and Institute for Life Sciences, University of Southampton, Southampton, UK; 4 Southampton NIHR Biomedical Research Centre, University Hospital Southampton Foundation NHS Trust, Southampton, UK; 5 Wessex Investigational Sciences Hub, Faculty of Medicine, University of Southampton, Southampton General Hospital, Southampton, UK

**Keywords:** *Haemophilus influenzae*, *Moraxella catarrhalis*, COPD, Exacerbation, Culture, PCR, Bacterial identification, Vaccination

## Abstract

**Background:**

Infection with *Haemophilus influenzae* (Hi) or *Moraxella catarrhalis* (Mcat) is a risk factor for exacerbation in chronic obstructive pulmonary disease (COPD). The ability to predict Hi- or Mcat-associated exacerbations may be useful for interventions developed to reduce exacerbation frequency.

**Methods:**

In a COPD observational study, sputum samples were collected at monthly stable-state visits and at exacerbation during two years of follow-up. Bacterial species (Hi, Mcat) were identified by culture and quantitative PCR assay. Post-hoc analyses were conducted to assess: (1) first Hi- or Mcat-positive exacerbations given presence or absence of Hi or Mcat at the screening visit (stable-state timepoint); (2) first Hi- or Mcat-positive exacerbations given presence or absence of Hi or Mcat at stable timepoints within previous 90 days; (3) second Hi- or Mcat-positive exacerbations given presence or absence of Hi or Mcat at stable timepoints within previous 90 days. Percentages and risk ratios (RRs) with 95% confidence intervals were calculated.

**Results:**

PCR results for analyses 1, 2 and 3 (samples from 84, 88 and 83 subjects, respectively) showed that the risk of an Hi- or Mcat-positive exacerbation is significantly higher if sputum sample was Hi- or Mcat-positive than if Hi- or Mcat-negative at previous stable timepoints (apart from Mcat in analysis 3); RRs ranged from 2.1 to 3.2 for Hi and 1.9 to 2.6 for Mcat.For all analyses, the percentage of Hi- or Mcat-positive exacerbations given previous Hi- or Mcat-positive stable timepoints was higher than the percentage of Hi- or Mcat-positive exacerbations if Hi- or Mcat-negative at previous stable timepoints. Percentage of Hi- or Mcat-positive exacerbations given previous Hi- or Mcat-negative stable timepoints was 26.3%–37.0% for Hi and 17.6%–19.7% for Mcat.

**Conclusions:**

Presence of Hi or Mcat at a stable timepoint was associated with a higher risk of a subsequent Hi- or Mcat-associated exacerbation compared with earlier absence. However, a large percentage of Hi- or Mcat-associated exacerbations was not associated with Hi/Mcat detection at an earlier timepoint. This suggests that administration of an intervention to reduce these exacerbations should be independent of bacterial presence at baseline.

*Trial Registration* https://clinicaltrials.gov/; NCT01360398, registered May 25, 2011

## Background

Bacterial infections are an important risk factor for exacerbation in chronic obstructive pulmonary disease (COPD) [1]. Various studies suggest that *Haemophilus influenzae* (Hi) is the main bacterial pathogen associated with exacerbations, followed by *Moraxella catarrhalis* (Mcat) and *Streptococcus pneumoniae* [2–5]. There is also evidence that Hi and Mcat are frequent co-pathogens in COPD [6] and that interactive effects protect Hi from complement-mediated killing in vitro [7] and promote an increased resistance of biofilms to antibiotics and host clearance [8, 9].

An investigational adjuvanted multi-component vaccine is being developed to potentially reduce the frequency of moderate and severe acute exacerbations in COPD associated with Hi and Mcat, containing four surface proteins involved in the virulence mechanisms of both bacterial pathogens. A phase 1 study showed the investigational vaccine had an acceptable safety and reactogenicity profile and induced antigen-specific immune responses [10], and phase 2 studies are underway in adults with COPD. We have previously observed that an investigational Hi vaccine containing the same components but without Mcat protein had an estimated vaccine efficacy of 13.3% against moderate/severe exacerbations in a clinical trial of adults with COPD [11]. The ability to predict an Hi- or Mcat-associated exacerbation based on baseline presence or absence of either bacterial may be useful for identifying patients who may benefit most from an intervention such as vaccination against these pathogens, helping to maximize any reduction in exacerbation rate.

In the observational study, Acute Exacerbation and Respiratory InfectionS in COPD (AERIS), Hi and Mcat were detected by PCR in 53.9% and 19.6% of exacerbation-state sputum samples, respectively, and in 44.5% and 11.2% of stable-state samples, respectively, in one-year follow-up of 127 patients with COPD [2]. In this post-hoc analysis, we examined whether positivity for Hi or Mcat at the screening visit for the AERIS study was linked to a higher probability of being positive for Hi or Mcat at the first exacerbation. An additional goal was to evaluate whether Hi- or Mcat-positivity within 90 days before exacerbation was associated with positivity for Hi or Mcat at exacerbation.

## Methods

### Samples

This analysis was based on sputum samples collected from subjects aged 40–85 years with a confirmed diagnosis of moderate, severe, or very severe COPD [12, 13] participating in the AERIS study (ClinicalTrials.gov registration, NCT01360398). This prospective, observational cohort study was conducted between 2011 and 2014 at University Hospital Southampton, in accordance with the Declaration of Helsinki and Good Clinical Practice and was approved by the Southampton and South West Hampshire Research Ethics Committee (Research Ethics Committee reference number: 11/H0502/9). All subjects provided written informed consent. The study methods were described previously [2, 12, 14]. Subjects were followed on a monthly basis for 24 months in the stable state and reviewed within 72 h of onset of exacerbation symptoms by using daily electronic diary cards. Sputum samples, obtained by spontaneous expectoration or induced, were collected at least every month (at monthly stable-state visits and at exacerbation).

### Bacterial species identification

Bacterial species presence in sputum samples was identified by culture and quantitative PCR (qPCR) assay, as described previously [2]. Culture of sputum samples was in accordance with Public Health England’s UK Standards for Microbiology Investigations [15], with slightly modified bacterial identification steps. In parallel, frozen (− 70 °C) aliquots of dithiothreitol-treated sputum samples were tested in a central laboratory by real-time triplex qPCR assay to identify and quantify Hi*,* Mcat, and *S. pneumoniae*. Since the analyses described in this paper focus on Hi and Mcat, only the PCR methods for these two species are described below. Nucleic acids were extracted using the *MagNA Pure* 96 DNA and Viral NA Small Volume Kit (Roche Diagnostics, Risch-Rotkreuz, Switzerland), as per the manufacturer’s instructions. DNA fragments of each pathogen were amplified using the *TaqMan* Fast Advanced Master Mix (Life Technologies, MA, USA). Sets of primers and probes were designed from the conserved region of the lipo-oligosaccharide glycosyltransferase encoding gene (*lgtC*) of Hi [16] and the copB outer membrane protein encoding gene (*copB*) of Mcat [17]. Positivity thresholds for each PCR target, which were set at the limit of detection defined during characterization of the technical performance of the PCR assay, were 2000 and 15,000 copies/mL for Hi and Mcat, respectively. Bacterial isolates (up to 10 per sputum sample) initially identified as Hi by traditional bacteriological methods were retested using a molecular approach developed by GSK, the *lgtC*/P6 duplex real-time PCR, which targets *lgtC* and the outer membrane protein P6 encoding gene (*omp P6*) [18]. Hi species identification by assessing the presence of both *lgtC* and *omp P6* previously showed an agreement of 96.61% against ‘gold standard’ multigene phylogenetic trees [16, 19]. An absence of Hi was assumed to indicate *H. haemolyticus* presence, based on previous observations published by Murphy et al. [20]. *Haemophilus* species was further assessed by whole genome sequencing and additional markers analysis, including the presence/absence of the *lgtC* gene and copper-zinc superoxide dismutase (*sodC*) gene, to confirm the presence of *H. haemolyticus*, as described by Osman et al. [21].

### Statistical analysis

Results are presented for sputum samples from stable visits and exacerbation visits for the full cohort of 127 subjects (all enrolled subjects, Year 1 and Year 2) [14]. The full cohort included all subjects regarded by the investigator as eligible for study procedures and excluded those who withdrew consent at the first visit.

The first analysis used sputum samples that were tested for Hi and Mcat presence at the screening visit and samples tested for Hi and Mcat presence at the first exacerbation experienced during the study. Numbers of sputum samples that were positive for Hi or Mcat at the screening visit and, of these, numbers positive for Hi or Mcat at the first exacerbation were calculated, as well as numbers of Hi- or Mcat-negative sputum samples at screening and, of these, numbers positive for Hi or Mcat at first exacerbation. The proportions of sputum samples positive for Hi or Mcat at first exacerbation and exact 95% confidence interval (CIs) were then calculated for the two groups. To test whether the difference between the two proportions (proportion positive at screening versus negative at screening) was statistically significant, the equality test between two independent proportions was applied and the p-value obtained.

A second analysis used sputum samples tested for Hi and Mcat in any stable study visit that occurred within 90 days before the first exacerbation experienced by each subject and sputum samples from the first exacerbation that were tested for Hi and Mcat presence. Numbers of sputum samples that were positive for Hi or Mcat at any stable visit conducted within 90 days before the first exacerbation and, of these, numbers positive for Hi or Mcat at the first exacerbation were calculated. Numbers of sputum samples that were negative for Hi or Mcat at all stable visits within 90 days before the first exacerbation were calculated and, of these, numbers positive for Hi or Mcat at the first exacerbation. Proportions and exact 95% CIs of sputum samples positive for Hi or Mcat at first exacerbation were then calculated for the two groups. The equality test between two independent proportions was applied and the p-value obtained.

For the third analysis of second exacerbations that were positive for Hi or Mcat given Hi or Mcat presence/absence at any stable visit within 90 days before the second exacerbation, the same process was applied as for the second analysis, substituting first exacerbation for second exacerbation. A sputum sample was considered positive within 90 days if at least one stable visit out of all stable visit records available within 90 days before exacerbation was positive for Hi or Mcat.

In each analysis, as well as calculating proportions, we calculated risk ratios for each comparative scenario. The risk ratio and its 95% CI were calculated using a generalized estimating equation (GEE) model with Poisson distribution and accounting for repeated observations. It has been shown that, when a Poisson model is fit to binary data (as in the present analysis), accounting for repeated observations provides robust variance estimators, which in turn lead to a proper estimate of the standard error of the risk ratio [22].

## Results

**Fig. 1:**
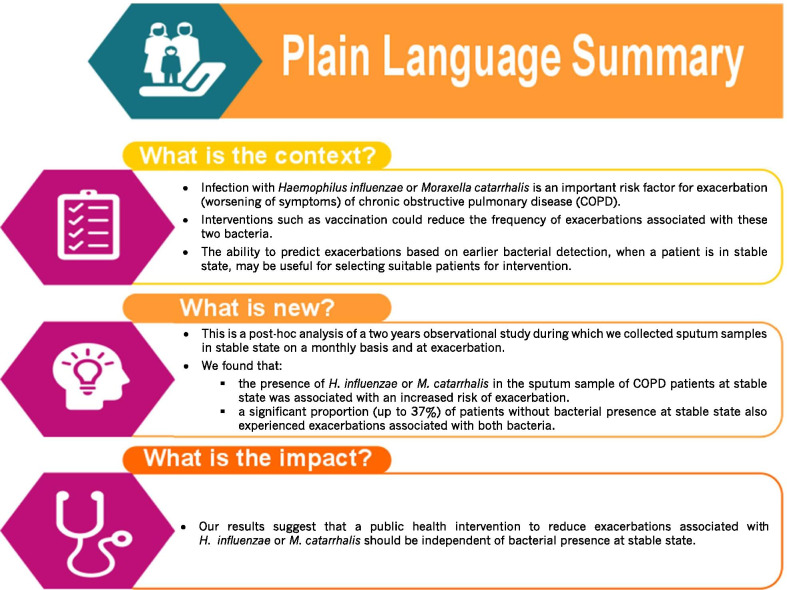
Plain language summary

Figure 1 summarizes the research, clinical relevance and impact of this study on the patient population.

**Fig. 2:**
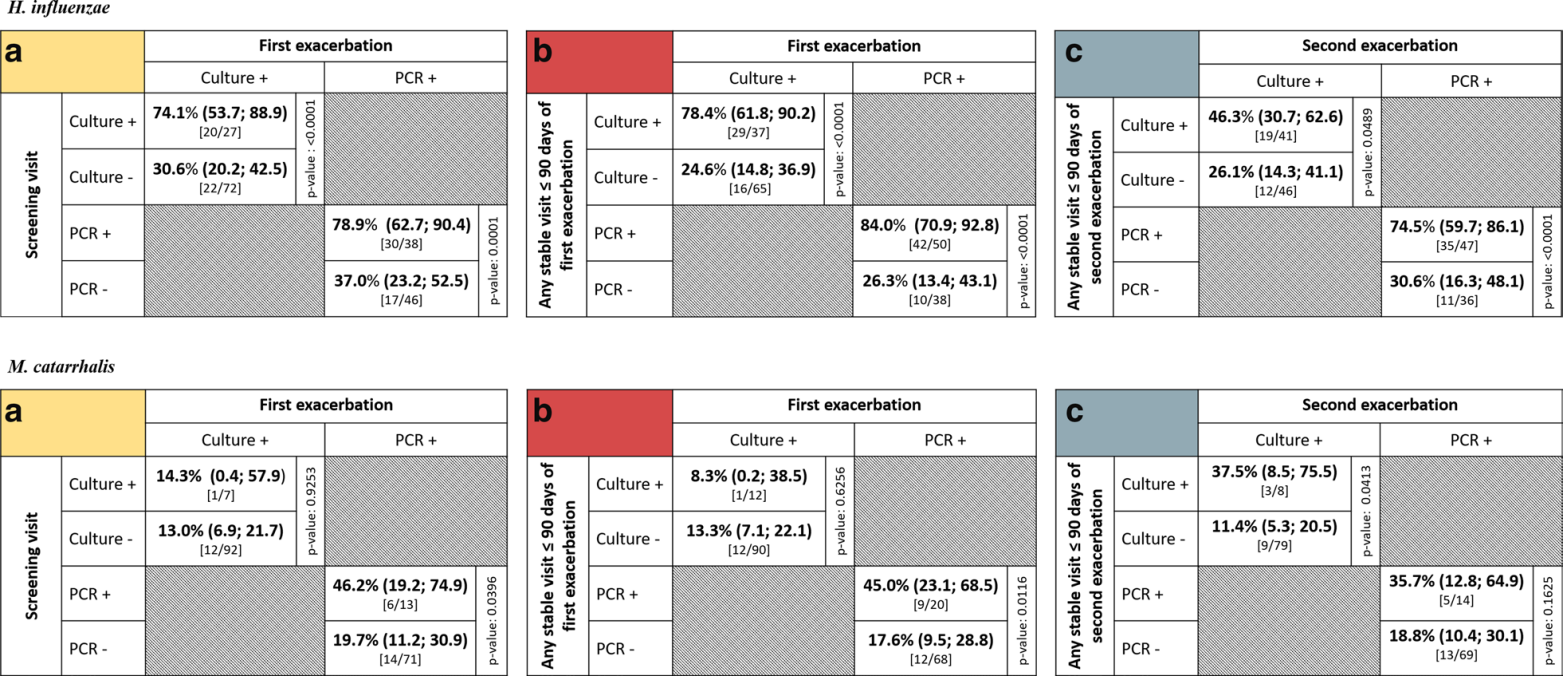
Positivity of first or second exacerbation by bacterial presence or absence at reference timepoint. Shown as percentage (95% CI) of samples that were positive for *Haemophilus influenzae* or *Moraxella catarrhalis* at first or second exacerbation given pathogen’s presence or absence at **a** the screening visit, **b** any stable visit within 90 days before the first exacerbation, or **c** any stable visit within 90 days before the second exacerbation (Full cohort, Year 1 and Year 2). 95% CI, 95% confidence interval; Culture +/− , *H. influenzae*- or *M. catarrhalis*-positive/negative by culture-based assay; PCR +/−, *H. influenzae*- or *M. catarrhalis*-positive/negative by PCR assay; p-value, p-value for difference between percentages, calculated by equality test between two independent proportions. Numbers in square brackets indicate the number of sputum samples positive at exacerbation/number positive or negative at reference timepoint

**Fig. 3:**
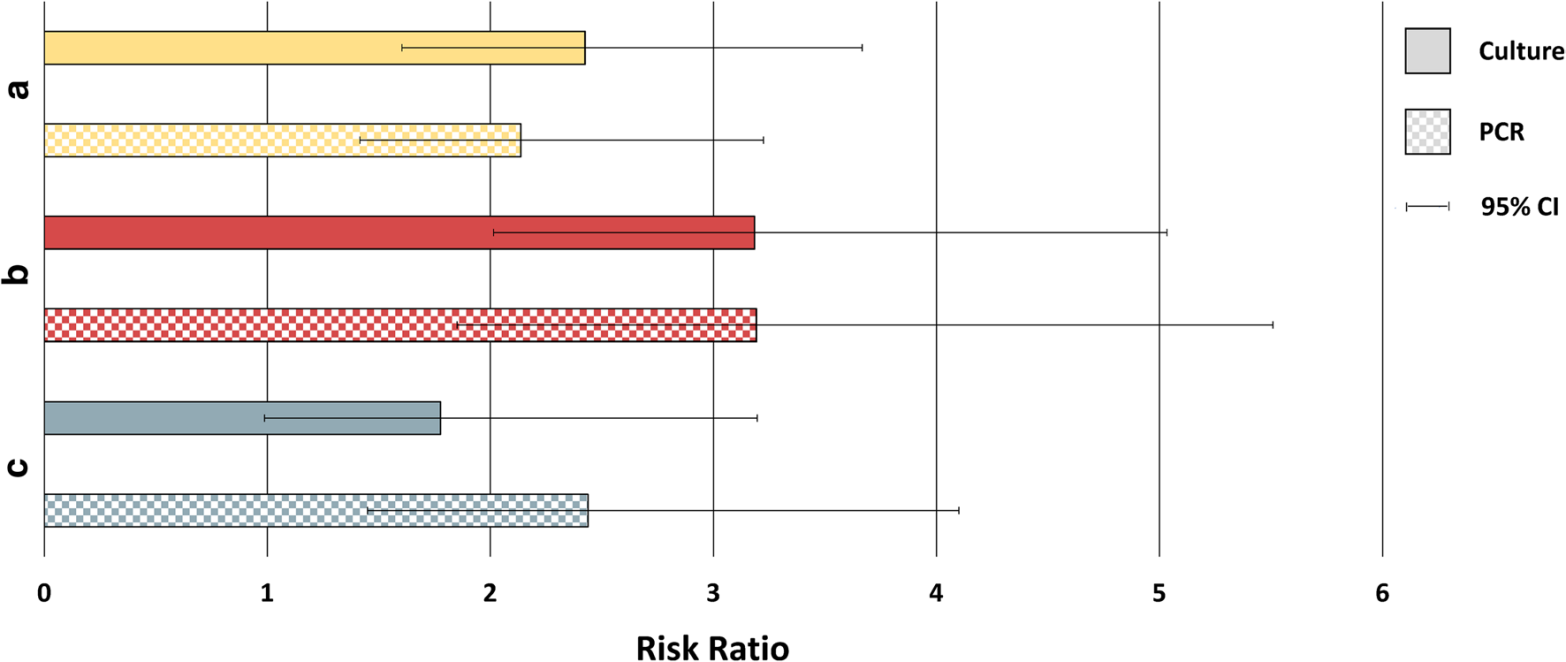
Risk ratio values for *Haemophilus influenzae* presence at exacerbation. Shown as risk ratio for *H. influenzae* presence at first exacerbation, given *H. influenzae* presence or absence at **a** the screening visit or **b** any stable visit within 90 days before the first exacerbation. **c** Risk ratio for *H. influenzae* presence at second exacerbation given *H. influenzae* presence or absence at any stable visit within 90 days before the second exacerbation (Full cohort, Year 1 and Year 2). **a** p-value < 0.001 for culture and PCR; **b** p-value < 0.0001 for culture and PCR; **c** p-value < 0.001 for PCR. Other p-values not statistically significant. 95% CI, 95% confidence interval

**Fig. 4:**
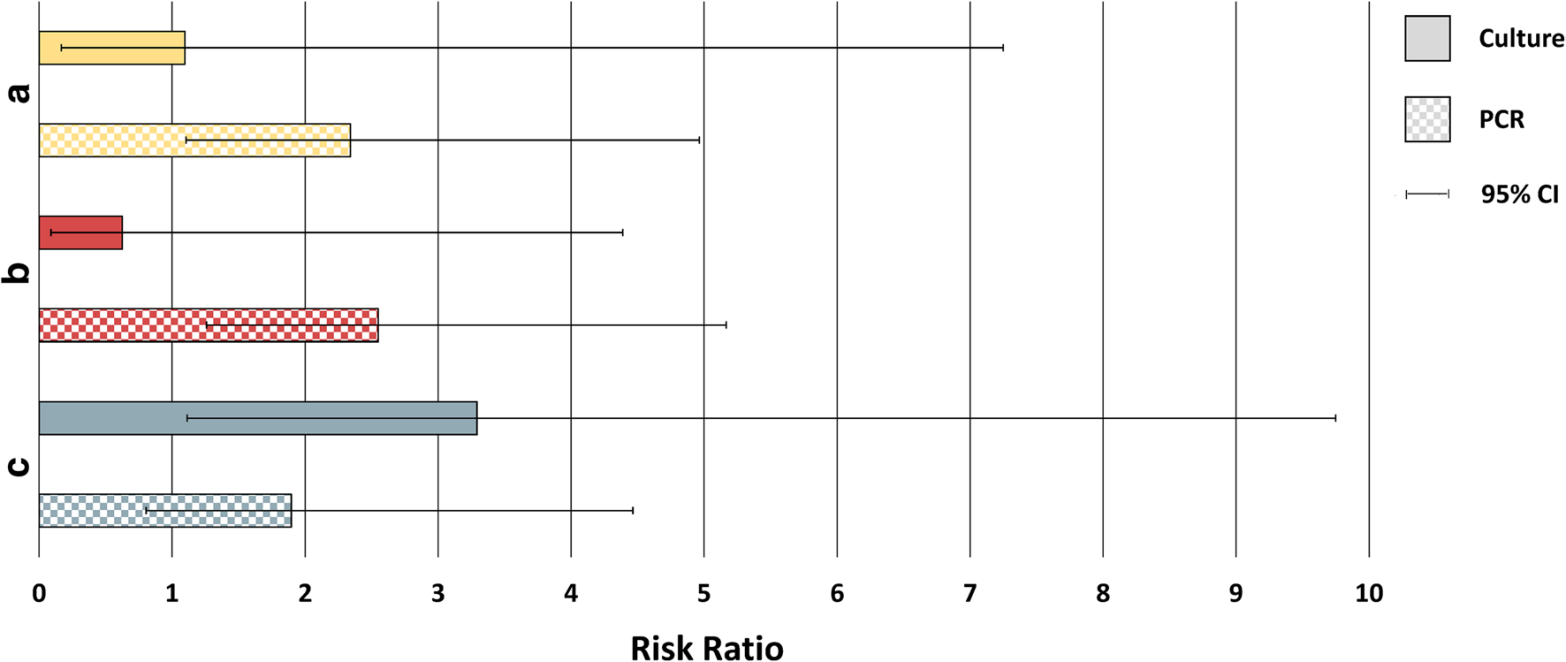
Risk ratio values for *Moraxella catarrhalis* presence at exacerbation. Shown as risk ratio for *M. catarrhalis* presence at first exacerbation, given *M. catarrhalis* presence or absence at **a** the screening visit or **b** any stable visit within 90 days before the first exacerbation. **c** Risk ratio for *M. catarrhalis* presence at second exacerbation, given *M. catarrhalis* presence or absence at any stable visit within 90 days before the second exacerbation (Full cohort, Year 1 and Year 2). **a** p-value = 0.03 for PCR; **b** p-value < 0.01 for PCR; **c** p-value = 0.03 for culture. Other p-values not statistically significant. 95% CI, 95% confidence interval

In the first analysis, the presence or absence of Hi or Mcat in the sputum sample from the screening visit was assessed in relation to Hi- or Mcat-positive sputum samples from the subject’s first exacerbation during the study. Sputum samples were available from 99 subjects for the culture analysis and 84 subjects for the PCR analysis. In the culture analysis, 74.1% of samples were positive for Hi by culture at both screening and first exacerbation, while 30.6% of samples were negative for Hi at screening but positive at first exacerbation (p < 0.0001) (Fig. 2). For the same comparison by PCR analysis, the percentages were 78.9% versus 37.0% (p = 0.0001). In the culture analysis, 14.3% of samples were Mcat-positive by culture at both screening and first exacerbation and 13.0% were Mcat-negative at screening and positive at first exacerbation (p = 0.925) (Fig. 2). For the same comparison by PCR, the percentages were 46.2% versus 19.7% (p = 0.040). Risk ratios showed the risk of being Hi-positive at first exacerbation given Hi positivity at screening was 2.4 (95% CI: 1.6, 3.7) times higher than for those that were Hi-negative at screening, as detected by culture (Fig. 3). With PCR detection, this risk was 2.1 (95% CI: 1.4, 3.2) times higher. The risk of being Mcat-positive at first exacerbation given Mcat positivity at screening was, by culture, 1.1 (95% CI: 0.2, 7.2) times higher and, by PCR, 2.3 (95% CI: 1.1, 5.0) times higher than for Mcat-negative samples at screening (Fig. 4).

In the second analysis, the presence of Hi or Mcat in at least one sputum sample or its absence in all samples obtained from stable visits within 90 days before the first exacerbation was assessed in relation to presence in the subject’s first exacerbation sample. Sputum samples were available from 102 subjects for the culture analysis and 88 subjects for the PCR analysis. The percentage of samples that were positive for Hi at both timepoints was 78.4% by culture and 84.0% by PCR versus 24.6% and 26.3%, respectively, for samples negative for Hi within 90 days but positive at first exacerbation (p < 0.0001, both analyses) (Fig. 2). For samples positive for Mcat at both timepoints, percentages were 8.3% by culture and 45.0% by PCR versus 13.3% and 17.6%, respectively, for samples negative within 90 days and positive at first exacerbation (p = 0.626, culture; p = 0.012, PCR). The risk of being Hi-positive at first exacerbation given Hi positivity within the previous 90 days was 3.2 (95% CI: 2.0, 5.0) times higher than for those that were Hi-negative within the previous 90 days, as detected by culture (Fig. 3). With PCR detection, this risk was 3.2 (95% CI: 1.9, 5.5) times higher. For Mcat, the risk of being positive at first exacerbation for those that were positive within the previous 90 days was 0.6 (95% CI: 0.1, 4.4) times higher by culture and 2.6 (95% CI: 1.3, 5.2) times higher by PCR than for those that were Mcat-negative within the previous 90 days (Fig. 4).

The third analysis was of second exacerbations that occurred during the study and were positive for Hi or Mcat, which were assessed in relation to the presence of Hi or Mcat in at least one stable visit or absence in all stable visits within 90 days of the second exacerbation. Sputum samples were available from 87 subjects for the culture analysis and 83 subjects for the PCR analysis. The percentage of samples that were Hi-positive at both timepoints was 46.3% by culture and 74.5% by PCR versus 26.1% and 30.6%, respectively, for samples negative for Hi within 90 days but positive at second exacerbation (p = 0.049, culture; p < 0.0001, PCR) (Fig. 2). Percentages for Mcat-positive samples at both timepoints were 37.5% by culture and 35.7% by PCR versus 11.4% and 18.8%, respectively, for samples negative within 90 days and positive at second exacerbation (p = 0.041, culture; p = 0.163, PCR). The risk of being Hi-positive at second exacerbation given Hi positivity within the previous 90 days was 1.8 (95% CI: 0.99, 3.2) times higher than for those that were Hi-negative within the previous 90 days with culture detection (Fig. 3). With PCR, this risk was 2.4 (95% CI: 1.4, 4.1) times higher. The risk of being Mcat-positive at second exacerbation was 3.3 (95% CI: 1.1, 9.7) times higher by culture and 1.9 (95% CI: 0.8, 4.5) times higher by PCR for those that were Mcat-positive versus Mcat-negative within the previous 90 days (Fig. 4).

## Discussion

We used sputum samples from participants in the AERIS study to assess the association between Hi or Mcat detection at first or second COPD exacerbation and Hi or Mcat positivity at either the screening assessment or a stable visit within 90 days before exacerbation. We found that the presence of Hi or Mcat at a stable timepoint was associated with an increased risk of a subsequent Hi- or Mcat-positive exacerbation among subjects with COPD, suggesting baseline status was predictive of bacterial status at exacerbation. While responder rates may be optimized by selecting only Hi- or Mcat-positive patients for an intervention to reduce Hi- and Mcat-associated exacerbations (such as the investigational Hi-Mcat vaccine in development [10]), our results indicate that this baseline stratification approach would exclude patients who could benefit. This is suggested by the large proportion of subjects identified in our study as being Hi/Mcat-negative at an earlier timepoint who went on to experience an Hi- or Mcat-associated first exacerbation: 37% and 20% of samples were, respectively, Hi- and Mcat-negative by PCR at screening, and 26% and 18%, respectively, were negative in the previous 90-day period. There was a similar finding in the analysis of positive second exacerbations (31% and 19% negative within 90 days for Hi and Mcat, respectively). Moreover, since there is marked seasonality to infection patterns associated with exacerbations in COPD [2], there could be additional benefit in protecting patients over the winter season, irrespective of baseline bacterial status. Given this and other factors that influence exacerbation risk [23], our results suggest interventions should be tested in all those at risk of exacerbation, accepting a spectrum of likely impact against this heterogeneous disease.

The first analysis was a comparison of bacterial presence at screening and at first exacerbation, while the second and third assessed Hi or Mcat presence at any stable visit within 90 days before the first or second exacerbation and presence at first or second exacerbation. Therefore, the second and third analyses considered all stable visits that occurred within three months before the first or second exacerbation. The 90-day limit was used to capture more timepoints so that, in the event of all stable visits being Hi- or Mcat-negative, there was more certainty in the association between being negative at preceding timepoints and positive at exacerbation. The number of exacerbation-positive samples associated with a positive sample from the 90-day period might be expected to be higher than the number associated with a positive sample at screening, but we did not find this to be the case consistently. An assessment period longer than 90 days is unlikely to provide meaningful results because of temporal variability in bacterial infections. Mcat infections tend to display greater temporal variability than Hi infections in patients with COPD, with Hi often becoming a component of the microbiota [24], while Mcat tends to have a more acute pattern of infection [25]. Where at least one of the timepoints in the preceding 90 days was Hi-positive, the percentage of positive samples at first or second exacerbation was higher than that associated with negative samples at previous timepoints, similar to observations from the first analysis. This provided further evidence that Hi positivity at stable state increases the probability of being Hi-positive at a subsequent exacerbation. The pattern was similar in the PCR analysis of Mcat but less consistent for culture identification of Mcat, which is likely to have been due to the low number of culture-positive samples.

The third analysis was of bacterial presence or absence at any stable visit within 90 days before the second exacerbation and presence at the second exacerbation. This is relevant to the clinical scenario where bacterial detection may have been missed at first exacerbation. The results were similar to those from the analysis of first exacerbations in relation to the previous 90 days: the percentage of samples that were Hi-positive or Hi-negative by PCR within the previous 90 days and associated with an Hi-positive second exacerbation was 75% and 31%, respectively, with corresponding percentages for Mcat of 36% and 19%, respectively.

Overall, the number of sputum samples positive for Mcat was lower than the number positive for Hi. This was in line with previous studies indicating a higher prevalence of Hi than Mcat in COPD [26–28]. Also, detection rates differed with culture versus PCR methods, with percentages generally lower and somewhat less consistent with culture-based detection. Other analyses of AERIS study results also showed higher prevalence of Hi and Mcat with PCR than with culture [2], which is in line with studies that showed better specificity and sensitivity with molecular techniques than with culture-dependent methods in the detection of airway bacteria in COPD [26, 29–31]. This provides further support for the use of well characterized molecular methods for the identification of bacteria in future studies of COPD.

Interpretation of the results of these analyses is limited by their post-hoc nature and the small number of samples, particularly Mcat-positive samples. Moreover, the study was not designed to predict if bacterial identification or bacterial load during exacerbation was causative or represented colonization. Also, it cannot be excluded that some samples that were negative by culture-based methods but positive on PCR assay contained viable bacteria that were not culturable, as reported in other studies [32, 33], or that there were non-viable bacteria present in samples recorded as positive by PCR [34]. However, it is unlikely that these factors had a significant effect on the trends observed.

## Conclusions

The presence of Hi or Mcat at a stable timepoint was associated with an increased risk of an Hi- or Mcat-related exacerbation. This suggests that earlier status predicts bacterial status at exacerbation and that response rates to a targeted intervention may be optimized by selecting Hi- or Mcat-positive patients. However, we found that a large proportion of subjects without baseline presence also experienced an Hi- or Mcat-associated exacerbation. Exclusion of this Hi- or Mcat-negative group may reduce the overall impact of an intervention given that a proportion could be expected to respond. These results support the implementation of an intervention, such as the investigational Hi-Mcat vaccine, independently of bacterial presence at baseline.

## Data Availability

Anonymized individual participant data and study documents can be requested for further research from http://www.clinicalstudydatarequest.com.

## Abbreviations

AERIS: Acute Exacerbation and Respiratory InfectionS in COPD
CI: Confidence interval
*copB*: CopB outer membrane protein encoding gene
COPD: Chronic obstructive pulmonary disease
GEE: Generalized estimating equation
Hi: *Haemophilus influenzae*
*lgtC*: Lipo-oligosaccharide glycosyltransferase encoding gene
Mcat: *Moraxella catarrhalis*
*omp P6*: Outer membrane protein P6 encoding gene
qPCR: Quantitative PCR
*sodC*: Copper-zinc superoxide dismutase

## Trademarks

*MagNA PURE*: Trademark owned by or licensed to Roche.
*TaqMan*: Trademark owned by or licensed to Roche Molecular Systems, Inc
